# *On Tic Douloureux:* "The Painful Affection of the Face, *Dolor Faciei Crucians*," of Fothergill, with a New Operation for Its Cure

**Published:** 1860-04

**Authors:** J. M. Carnochan

**Affiliations:** Surgeon in Chief to the State Hospital, &c.


### SELECTED ARTICLES.

### ARTICLE XII.

*On Tic Douloureux:* "The painful affection of the face, *Dolor Faciei Crucians,*" of Fothergill, with a new operation for its cure. By J. M. Carnochan, Surgeon in Chief to the State Hospital, &c. Read before the Medico-Chirurgical College, January 12th, 1860.

Some months ago I published a paper on *Tic Douloureux,* or neuralgia of the face, and at the same time proposed an operation for the cure of the disease, which in my opinion is founded upon the physiological laws which govern the functional manifestations of that part of the nervous system presiding over general sensation, as well as upon certain pathological appearances, which I had found to be present in portions of nervous trunks after their exsection from the face. I propose this evening to bring before the "College" the views I have heretofore stated in relation to the pathology, seat and treatment of neuralgia of the face, and to describe my latest operation for exsection of the trunk of the second branch of the fifth pair of nerves, as far as the foramen rotundum of the sphenoid bone—an operation which I believe to be an improvement on the one I at first proposed for the exsection of that nerve. Before I proceed, permit me to congratulate myself upon the presence of so large a number of gentlemen, whose knowledge of physiology will insure a close analysis of the remarks which I shall have the honor to make.

Let us for a moment glance at the amount of precise information which science had afforded on this subject up to the time I published my paper some few months ago.

It is only within a comparatively recent period that neuralgia of the face has attracted the serious attention of medical authors; or, perhaps, it may be more properly stated that the disease has been surrounded by such obscurity and perplexity, as to have baffled description. André, a French surgeon, in 1756, published his researches upon this malady, and gave to it the name of tic douloureux. According to this writer, the affection is characterized, "*par une douleur plus ou moins vive, et par des grimaces hideuses qui mettent un obstacle invincible à la réception des aliments, qui, éloignent le sommeil, interceptent et lient souvent l'usage de la parole: agitations qui, quoique vagues et périodique en elles-même, sont néanmoins si fréquentes, quelles se font sentir plusieurs fois dans un jour, dans un heure, et quelque fois sont sans relâche et se renouvellent à chaque minute.*" This description, as far as it goes, refers to the disease when it has acquired a high degree of intensity, and coincides with the malady which Fothergill soon after (in 1782) described under the name of "Painful Affection of the Face." The account given by Fothergill is more complete and exact than that of the French surgeon just named, as we may learn from the following extract:

"From imperceptible beginnings, a pain attacks some part or other of the face, or the side of the head; sometimes about the orbit of the eye, sometimes the *ossa malarum*, sometimes the temporal bones are the parts complained of. The pain comes suddenly, and is excruciating; it lasts but a short time, perhaps a quarter or half a minute, and then goes off; it returns at irregular intervals, sometimes in half an hour, and sometimes there are two or three repetitions in a few minutes.

"The kind of pain is described differently by different persons, as may be reasonably expected; but one sees enough to excite one's compassion, if present during the paroxysm.

"It returns full as often in the day as in the night. Eating will bring it on some persons. Talking, or the least motion of the muscles of the face affects others; the gentlest touch of a hand or handkerchief will sometimes bring on the pain, whilst a strong pressure on the part has no effect."

About the same time that Fothergill's memoir appeared, Thouret, another French writer, published an article on this subject, in the *Mémoires de la Société Royale de Médicine.* This writer states that the disease appears to fix itself particularly in certain localities of predilection: "*comme la mâchoire inférieure, le trou mentonnier, le voisinage de l'apophyse mastoïde et la région de la joue la plus voisine de l'œil. En général,*" he adds, "*le siège le plus ordinaire du mal est sur le côté du nez immediatement au-dessous de l'os de la pommette, à l'endroit ou une branche principale du nerf maxillaire supérieur sort du canal sous-orbitaire.*"

The authors heretofore alluded to, contributed, by their observations, chiefly to the symptomatology of this disease.

Chaussier, guided by his anatomical knowledge, made some steps of advancement in determining the seat of neuralgia of the face. In his "*Table Synoptique de la Néuralgie,*" (Paris, an XI,) he makes four principal divisions of neuralgia of the face, according as the disease seems to be concentrated upon one or other of the branches of the fifth pair of nerves, or upon the facial nerve proper, itself. Thus he describes separately *frontal neuralgia, infra-orbital neuralgia, maxillarg neuralgia;* and in a note he admits the existence of neuralgia of the *facial nerve.* The course of the tri-facial nerve and its anastomoses, have evidently formed a basis of this classification.

Since the publication of Chaussier, several dissertations and theses have appeared at different times, in which new cases are related, but without the addition of any novel information on the subject.

In 1834, M. F. Bellingeri, of Turin, published a memoir upon neuralgia of the face; the chief features of which are his advocacy of an intermittent form of neuralgia, and the theoretical division of neuralgia into three species—the *inflammatory*, the *irritative*, and the *nervous*; the first species being again subdivided into the *sanguine*, the *phlogistic*, and the *rheumatismal*.

In more recent times, (1835–36,) the question has been agitated by M. Bérard, as to the existence of a neuralgia of the *facial nerve* proper. This physiologist has arrived at the conclusion that the nerves of the *fifth pair* alone, in the face, can be attacked with this malady. Upon the other hand, in a later publication, M. Jobart de Lamballe supports an entirely different opinion, and maintains that all the nerves of the face are liable to be attacked by neuralgia. In regard to this last point of discussion, we are aided in arriving at a correct conclusion by anatomical facts, as well as by the morbid phenomena which are frequently exhibited in neuralgia of the face. The *portio-dura*, or facial nerve, is undoubtedly a nerve purely of motion, at its origin; but, before it emanates from the stylo-mastoid foramen, it has been joined by a sensitive branch from the ganglion of Meckel. Physiologically, after this junction, the facial nerve must be a mixed nerve, and must be sentient to impressions, both normal and abnormal. In fact, I consider that the facial nerve is not only the seat of neuralgia at times, but that it is frequently the conductor of neuralgic phenomena, and morbid sensibility, to the nervous periphery of the face, when the true seat of the disease is located on the trunk of the second branch of the fifth pair. I make this statement, partly from observation, and partly from the consideration of the law which regulates the propagation of nervous sensibility, viz. that the sensation is referred to the periphery of a nerve, or to its extreme branches, when the trunk is the seat of irritation or disease. A simple illustration of this is found in the effects which follow a blow impinging upon the ulnar nerve at the elbow. It is well known that the impression imparted to the trunk is conducted to the ultimate distribution of the nerve, and that the sensation is referred to the little finger, and to the ulnar border of the ring finger.

By reference to the history of this disease, we find that notwithstanding its formidable character, and the numerous attempts made to unravel its pathology, science has not been as much enriched as might be supposed, with facts observed with care and precision. With the exception of the discoveries of Charles Bell, and others, in relation to the functions of the nerves, which are implicated in neuralgia of the face, and some vague and unsatisfactory therapeutic experiments, we have made but little advancement in regard to the nature and management of this disease, since the time of Fothergill.

As yet authors differ concerning the anatomical lesions which should be considered as characteristic of neuralgia of the face. Some deny that any are to be found in those cases where the signs of the disease have been indicated in an incontestable manner; and one of the latest writers expresses himself in the following indefinite language: "*La neuralgie tri-faciale doit être considérée comme une lesion de fonction donc nous ignorons entièrement la cause organique.*"

Again, the seat of the disease has been referred to distant irritations, especially in the splanchnic cavities—to a foreign body acting upon the nerve—to the pressure of bone upon some portion of the nervous trunks. By some authorities, it is referred to increased vascularity and thickening of the nerves; while Astley Cooper, on the contrary, states, that the nerves present their natural color, and are rather diminished in size than enlarged.

The medical treatment, although embracing a vast number of therapeutic agents, is most frequently unavailing; and it is well known that the surgical treatment, consisting principally of topical applications, and the division on the face of the branches of the fifth pair, at their exit from their respective foramina, is, for the most part, utterly useless in affording relief, or at best affords but very temporary benefit. The medical inquirer, then, has no reason to be satisfied with the observations heretofore made, but, on the contrary, is justified and urged in bringing under scientific scrutiny such new facts as may assist in unveiling still further the mysteries which envelop this most painful malady.

In Part Two of my "Contributions to Operative Surgery and Surgical Pathology," I have described a case of Neuralgia of the Face, certainly the most remarkable on record for its severity and protracted course, and for the number of operations which were performed on the patient. The facts which were developed during the different stages of the treatment led me to project an operation for the cure of aggravated *Tic Douloureux.* It had for its object the exsection of the trunk of the second branch of the fifth pair, beyond the ganglion of Meckel; at the same time removing that ganglion, or insulating it with its branches from the encephalon. Experience, however, has since convinced me that it is not adequate to effect the removal of the whole trunk, as far as the base of the skull, with the same certainty and safety from hemorrhage as that which I now adopt.

The following is the history of the case, and here standing before you is the patient himself, finally cured by an operation, which will hereafter be described. [The patient was presented and examined by the members present.]

*Case.*—J. C. Forbes, aged 47, a citizen of Hoboken, New Jersey, married, of nervo-bilious temperament, by occupation a master carpenter, applied to me for advice in the month of August, 1855. He had, with the exception of his neuralgic affection, always enjoyed good health, and had no special taint of the system.

He stated that he observed the first signs of this disease in May, 1849. At that time, while passing a handkerchief across the upper lip and end of the nose, he perceived a sharp, poignant, lancinating pain, shooting from near the middle of the upper lip, on the left side, along the furrow at the junction of the nose and cheek, up to the inner angle of the eye of the same side, and passing deeper through the bone of the cheek, in the direction of the spheno-maxillary fissure. The same pain was started when the upper lip was touched with the tip of the tongue, or when making an effort to swallow. These symptoms, assuming a paroxysmal character with irregular intermissions, continued unabated until the autumn of the same year, when they entirely disappeared.

In the spring of 1850, the attack was renewed, commencing as it had done the previous year, and gradually becoming more painful. It was supposed by some that the trouble might originate from disease of the teeth; and, by the advice of a dentist, all the teeth were extracted, except a small stump on the right side of the lower jaw. This proceeding gave no relief; the disease increased in severity; the paroxysms of pain became more frequent, and almost intolerable, extending over the entire left cheek.

From that time (June, 1850) until February, 1852, the patient continued under medical treatment; he gave up his business, and sedulously tried the most approved prescriptions; but in vain.

Finding no relief from the use of internal remedies as advised by skillful physicians, he consulted a hospital surgeon of eminence, in this city, with the view of having an operation performed, if deemed expedient. An operation was advised and performed (February, 1852) by dissecting, from the interior of the mouth, without external incision, the entire cheek from the superior maxillary bone; the separation of the tissues extending across from the nose to the prominence of the malar bone, and vertically, from the alveolar border, as high as the margin of the left orbit. A considerable quantity of blood flowed while the incisions were being made. Much relief followed this operation, and the paroxysms seemed to be kept at bay for a period of about seven months.

The following November, the disease returned with its wonted severity, and the patient was again put under medical treatment, using chiefly large doses of quinine. In the latter part of December, 1852, the paroxysms became so aggravated and intolerable, that the patient again entreated the same surgeon to perform another operation. This was accordingly done by making a V incision below the margin of the orbit, and dissecting the flap upward, so as to expose the infra-orbital foramen. The nerve was then divided at its exit upon the cheek. The patient was again relieved, until the autumn of 1853, when the pain returned, with severer manifestations than before. The patient was again put under medical treatment, galvanism being added, without any advantage, to the long list of therapeutic means previously resorted to.

In January, 1854, a professor of surgery, of some eminence in this city, was consulted, and performed the same operation as that last described; but besides the incisions for dividing the nerve, he cauterized with a red-hot iron the divided surface of the nerve, at the infra-orbital foramen. Relief was again obtained until August of the same year. During that month the paroxysms reappeared with their previous intensity, and another operation of a similar character was performed in September, by another surgeon, also of this city; but without any good result.

In October of the same year, (1854,) harrassed, despondent, and worn out with the accumulated violence of his sufferings, the patient consulted my celebrated friend, Professor Mott. With the hope of affording relief, the tissues of the cheek were freely divided by subcutaneous incision, once in October, and again in November, by that distinguished surgeon. Very slight amelioration of the pain was effected by these operations, and on the 8th of January, 1855, Professor Mott performed his third operation, by making a V incision on the cheek, in the same manner as had been previously done, and dividing again the nerve at the infra-orbital foramen.

These numerous operations, although lulling the terrible sufferings for a time, left no lasting impression on the disease. For nearly five months a partial mitigation of the symptoms followed Dr. Mott's last operation, but in June, 1855, the pain set in afresh with its accustomed violence; and the patient, unable to attend to bnsiness, his means exhausted, his strong frame shattered with prolonged and intense agony, and his mind paralyzed with despair, took refuge in the New York City Hospital. There he remained in the medical department, under the care of the physicians of that institution, until the month of August, 1855, when he took his discharge; having received no benefit from the treatment prescribed.

It was at this stage of the malady that the patient came under my notice. A few days after he left the hospital, he was brought by some of his friends to consult me, in a condition bordering on delirium: wild, and almost mad, as he himself stated, with the intensity of his sufferings. He most piteously besought me to perform some operation for him, different from those previously tried; protesting that he was utterly regardless of any danger that might be incurred, or of the extent or character of the mutilation which might result.

After hearing a recital of the various operations to which he had been subjected, and seeing, from his condition, that nothing really useful had been accomplished, it seemed to me futile and hopeless to recommend an operation which could consist of the division simply of the nerve. As the catalogue of medicines of most repute in neuralgia had been exhausted unavailingly, it was useless to repeat them, and ordering, for the moment, a strong dose of muriate of morphine internally, and an ointment composed of the extracts of belladonna, hyoscyamus, and stramonium, with a small portion of veratria, I undertook to perform on him, on the following day, the operation of exsecting a piece of the trunk of the infra-orbital nerve.

The condition of the patient, after his return home from the hospital until he came to me for advice, was truly appalling, and enlisted the sympathies even of strangers. He could neither rest, sleep, eat, drink, nor talk, without the occurrence of paroxysms of the most violent character. He would start from his couch with the wildness of a lunatic, and would throw himself on the floor, screaming and howling from the intensity of his agony. At times, his sufferings would overcome his moral courage, great as that was, and he would threaten self-destruction. The slightest impressions on the external surface of the face, especially on the upper lip or cheek, or upon the mucous lining of the mouth, pharynx, or nose, would bring on, most commonly, paroxysms of the most aggravated description. The intermissions between the paroxysms, during the exacerbations or attacks of the disease, were variable; sometimes lasting half an hour or more, sometimes a few minutes, and at others only some seconds.

On the 31st of August, 1855, I performed the following operation on Forbes: He was seated on a chair in a good light, and the assistants being properly arranged, he was put under the full influence of chloroform. A V-shaped incision was made, the base towards the margin of the orbit, and embracing the infra-orbital foramen. The flap thus formed was dissected upward to the margin of the orbit, and the dissection was extended still further, so as to expose half an inch of the osseous floor of the orbit. There was some difficulty in finding the foramen, and in insulating the nerve, owing to the matting of the tissues, as well as to the extensive and hard cicatrices, resulting from the previous operations, performed at the same point. The nerve, at its exit from the foramen, being found with the hammer and chisel, a portion of bone was detached from the margin of the orbit, so as to remove the upper semi-circumference of the foramen infra-orbitale. Another piece of bone was now easily removed from the anterior part of the infra-orbital canal, and a portion of the trunk of the nerve was thus exposed. The nerve was easily detached from the canal at this part, and about a quarter of an inch of its trunk was exsected rapidly with the scissors.

Although chloroform was very freely administered, it was found almost impossible to keep up the anæsthetic influence sufficiently to annul the pain. While apparently insensible, when the nerve was touched with the instruments he would start violently, uttering a fearful shriek, and would become almost immediately perfectly conscious.

This operation by excision effected a greater degree of immunity from the severe symptoms than had been afforded by the mere division or incision of the nerve. Up to this period, from the time of the first attack, the patient had not been able to eat or drink without starting the severe pain in the face; the neuralgic paroxysms now, however, were not incited by the act of swallowing. In fact, the relief was immediate, and the disease seemed to be cured.

This relief from suffering was not of long duration. In February, 1856, the paroxysms were again renewed with as much intensity as ever, and the patient again demanded another operation. His condition was one of desperation, and justified a resort to any means which held out the slightest probability of success. I proposed to him, explaining the nature of the operation, to lay open his face, trepan the antrum maxillare, separate the trunk of the second branch of the fifth pair from its connections, as far as the posterior part of the antrum, and then to exsect a still larger portion of the nervous trunk. An eager and ready assent was given to this suggestion, and on the 21st of February, 1856, I accomplished the operation in the following manner: An incision was made, commencing opposite the lower border of the left orbit, below the inner angle of the eye, and carried downward and outward, so as to terminate at a point about half an inch below the infra-orbital foramen; another incision, beginning at the lower border of the orbit, and below the outer angle of the eye, was made so as to join the lower extremity of the first. At the junction of these two incisions, a sharp pointed straight bistoury was now thrust through the cheek, and the upper lip divided entirely, midway between the median line and the labial commissure. The V-shaped flap first made, was dissected upward, and the other flaps were thrown inward, towards the nose, and externally over the malar bone. The fore part of the antrum maxillare and lower margin of the orbit were thus freely exposed. The situation of the foramen infra-orbital was easily ascertained, and the crown of a trephine, three-quarters of an inch in diameter, was applied upon the anterior wall of the antrum ; the trephine grazing the lower border of the foramen. A portion of bone was thus removed, so as to expose the cavity of the antrum. The membrane lining this cavity was found to be thick and velvety, and to present a dark maroon color. The anterior portion of the trunk of the nerve was sought for and found. With a hammer and delicate chisel, the infra-orbital canal was laid open, and the nerve detached from its bony wall, as far backward as the posterior wall of the antrum. The operation was finished by exsecting, by a rapid movement of the scissors, to the extent of about an inch, the nervous trunk thus laid bare and exposed. A pledget of dry lint was placed in the antrum, and the various incisions brought together by means of the Carlsbad pins as sutures.

Notwithstanding the complete anæsthesia under which the patient was kept, he would frequently, when the trunk of the nerve was touched with the forceps or chisel, jump from the chair as if struck by a powerful shock of electricity.

After this operation the unfavorable symptoms again disappeared, and the patient flattered himself that a cure had been effected. The paroxysms, with the exception of an occasional *tic*, abated, and he had such confidence in his recovery, that he accepted an offer to visit Panama, New Granada, to engage again in business.

On the 20th of September, 1856, he left the United States, and arrived at the Isthmus of Panama during the following month. Six months were passed there without any annoyance from the disease. In the month of March, 1857, after exposure to cold, and sleeping in a damp atmosphere, the pain again appeared with much severity. It now seemed to commence from a point in the upper maxilla, opposite the alveolar border, where the first incisor tooth had been extracted, and to dart backward with great acuteness towards the spheno-maxillary fossa. The paroxysms of pain were as severe, although not so diffused, as those by which the disease was ushered in.

Forbes was again forced to relinquish his business, and sailed for New York city, where he arrived in the latter part of April, 1857. He paid me a visit, and related to me the story of his recent attack, with an air and expression of utter despondency. In fact, he was again laboring under all the symptoms of tic douloureux.

On the 20th of April, at his earnest request, I performed another operation on him. This consisted in dissecting back the tissues of the cheek, and exposing the antrum maxillare. By the use of Lüer's bone-cutters, I cut away the outer and lateral wall of the antrum, as low down as the alveolar margin of the bone, so as to destroy the loop of nerves which results from the anastomoses between the branches of the posterior and anterior dental nerves. This operation (the tenth) was of very little avail.

During the following three months he resorted to the free use of narcotics, for the purpose of annulling the pain. In September, 1857, Forbes again entreated me to do something for his relief, and I dissected the cheek from the bone, by dividing the cicatrices which had been recently formed. This afforded temporary relief, resulting probably from the local depletion.

The cheek and left upper lip were now insensible to the touch, and the spasms were aroused by eating or swallowing; or they occurred spontaneously. The pain was referred, chiefly to the upper maxillary bone, commencing at the point where the two left incisors had been extracted, and darting backward in different directions, towards the base of the skull. A few days only of partial relief followed, when the pains were renewed with such severity that Forbes again besought another attempt to procure some amelioration of his suffering.

From the failure of the operation by which about an inch of the trunk of the second branch of the fifth pair of nerves had been removed, it occurred to me that the portion of the trunk which was left must be still in a diseased condition, and that the train of neuralgic phenomena which were manifested, was to be referred to the peripheric ramifications, emanating from the ganglion of Meckel, and also to those branches which emanated from the small portion of the trunk of the nerve still remaining in front of the *foramen rotundum.*

As Forbes' case at this time presented itself, I was unwilling to cut down again through the cheek and seek for the remaining stump of the nerve, in order to exsect this as well as the ganglion of Meckel. With a faint hope of mitigating the disease, on the 2d of October, 1857, I again laid bare the antrum and the bones of the cheek, by making, through the soft parts of the cheek and through the lip, the same incisions which had been made in my second operation for exsection of a portion of the trunk. With Lüer's bone forceps I then cut away the remaining portion of the anterior and lateral portions of the antrum, with a part of the posterior wall of this cavity; removing a part of the alveolar border of the upper maxilla, so as to encroach partially upon the vault of the mouth; while toward the nose, the portion of bone opposite the two left incisors was removed, as well as a considerable portion of the ascending process of the superior maxilla. By this operation it was intended to destroy still further the several nervous branches running through the texture of the bones of the face, on the affected side. A dossil of lint was laid in the cavity of the wound, and the lips of the incisions brought together by the twisted suture. The wound healed, but a free communication remained from the mouth with the antrum, between the cheek and the edge of the vault of the mouth.

As before, a cessation of the symptoms followed the operation. But the paroxysms returned in a few weeks, with tempered severity, however, and at longer intervals. When the pain did not start as if spontaneously, impressions made upon the nasal, buccal, or pharyngeal mucous membrane appeared to be the exciting or immediate source of the paroxysms. It is upon those surfaces that the peripheric extremities of the branches, which take their origin from the ganglion of Meckel, are distributed. In order, therefore, to change the sensibility of the mucous surface, I began to cauterize freely, upon alternate days, the mouth and pharynx, as well as the antrum and cavity of the nostril, with a strong solution of nitrate of silver, by injecting the solution into the antrum from the mouth, through the communicating passage now existing. By these means and the occasional use of narcotics, the patient obtained very great relief.

In addition to the action of the nitrate of silver upon the mucous surfaces just mentioned, on the 3d of January, 1858, a seton was introduced into the back of the neck, on the left of the mesial line, for the purpose of maintaining a continued revulsive influence in proximity to the fifth pair of nerves. From this time, also, ten grains of quinine were daily administered internally. During the twelve months following the last operation, (October, 1857,) Forbes had comparative immunity from his disease; occasionally, however, he would be attacked with sharp paroxysms of pain suddenly passing through the left side of the face. He had also returned partially to his business, and ate, drank, and slept with tolerable comfort.

This respite from suffering was interrupted on the 15th October, 1858, when he had, after exposure to cold, some severe and sharp paroxysms. These were excited principally by the act of swallowing either fluid or solid articles of food. After continuing for two days, the paroxysm ceased under the influence of a cataplasm of stramonium leaves, and the tincture of aconite administered internally. At the date of November 4th, 1858, he was so well that he was about to resume his business, and on the preceding Tuesday he went to the polls to deposit his vote as an elector.

Notwithstanding his ameliorated condition, it could not be said that the patient was cured. It was not improbable that he would be liable at times to be attacked with paroxysms of his disease.

As was partly expected, Forbes was again attacked with the disease, and with such severity that, in June, 1859, he called upon me and insisted upon being subjected to another operation. By this time, from repeated operations, I had come to the conclusion that the only effectual treatment was the exsection of the portion of the trunk still remaining in front of the foramen rotundum, in immediate connection with the ganglion of Meckel; thus insulating that ganglion and its branches from the encephalon. In accordance with this view, the remaining stump of the trunk was exsected, close to the foramen rotundum. The result was satisfactory, and continues to be so.

I shall now proceed to offer some general considerations. *Tic douloureux* of the face proper, or of the second branch of the fifth pair of nerves, is by far the most common form of facial neuralgia. This may be explained by the more numerous branches which are given off by this trunk, and by the position which these branches occupy—in some places, pent up in osseous canals, and in others, subjected to exposure, to changes in temperature, as well as to the agency of morbific influences, from which the other two trunks of the fifth pair are exempt.

I believe that the phenomena of this neuralgia can be explained with as much precision as in any other disease which is well understood. In cases of the most aggravated form, whatever may have been the original exciting cause, I have no doubt that the real seat of the disease is in the trunk of the nerve, in front of the *Gasserion ganglion*—in some part of it, or in the whole of it. The causes of the disturbed and changed condition of the trunks of the nerve may be numerous—prolonged irritation upon the periphery—exposure—injuries—tumors—diseases of the teeth—pressure resulting from periosteal or osteal thickening of the osseous foramina or canals—sudden suppression of any of the important secretions, as of the catamenial discharge. From one or more of these causes the trunk itself may be primarily affected, or, acting upon its ramifications, the irritation may be propagated to it. Prolonged irritation induces inflammation, and this generally remains passive or chronic. Some of the terminations of inflammation—such as the effusion of lymph among the interstices of the neurilemma or the nervous tissue itself—may become developed; leading to a vascular, engorged, thickened, and enlarged condition of the nerve, or to a softening of it, at one or more points. In fact, vascular engorgement, or inflammation, with some of its consequences, of the neurilemma alone, or of it and the nerve together, by whatever cause produced, is the condition which constitutes the pathological changes in the trunk.

In all cases where I have performed exsection, the nerve was found to be red, vascular, engorged and considerably enlarged.

The diffused character of the pain can be easily understood, if we take into consideration the numerous ramifications of the second branch of the fifth pair, and the extensive surface over which their ultimate filaments are distributed. The periphery of the nerve occupies not only the superficial parts of the face, but extends deep amongst the bones of the upper jaw, to the nasal fossæ, to the septum nasi, to the hard and soft palate, to the pharynx, to the inner ear, to the orbit, and to the temporal and malar regions.

It is well established, that if the trunk of a nerve be irritated along its course, the painful sensation will be referred to its periphery. If the ulnar nerve, for example, be struck where it passes behind the internal condyle, a sensation of pain is excited, which is referred to the little finger, and to the ulnar border of the ring finger ; and if a prolonged irritation be kept up at this point, the skin of these fingers becomes tender to the touch, the sensibility being very much increased. The pain which is felt at the knee, in *morbus coxarious*, also illustrates this law. "The obturator nerve," Sir Charles Bell remarks, "passes through the thyroid foramin, down to the hip-joint, and, after supplying the muscles, is distributed upon the inner part of the knee. The nerve in its course is thus involved in the inflammation which affects the hip-joint, and the pain is referred to its extreme cutaneous branches, at a part distant from the seat of the disease.

It is by this principle—which governs the action of the stimuli upon the nerves of sensation—in connection with the anatomical distribution of the nervous ramifications, that numerons phenomena of neuralgia can be explained. The disease being seated in the trunk of the nerve, we can readily understand that the pain must be referred to the peripheric extremities of the nerves, and will there be felt as long as the branches are in communication with the encephalon.

From these views we can see how futile the operation of division of the nerve at the *foramen infra-orbitale* must be. Where the trunk of the nerve is extensively diseased, no operation can rationally lead to a successful result, unless all the branches emanating from the trunk are cut off from communication with the brain.

I believe that in all aggravated cases of neuralgia, of the second branch of the fifth pair, the key of the operation is *the removal of the gangliou of Meckel, or its insulation from the encephalon.*

We can account for the return of the neuralgic pain, after exsection of a large part of the nervous trunk, by the induction, that some portion of the remaining nerve becomes again attacked by disease. It is well known that although the periphery of a nerve may be removed, yet, when the stump of a nerve is the seat of irritation, the person feels the pain at the locality of the former periphery. In this manner I account for the frequent return in Forbes' case of the neuralgic pain. The ganglion of Meckel, also, if left unremoved or not insulated, continues to provide, to a great extent, nervous ramifications, which will still maintain and keep up the diversified neuralgic pains. Besides, the ganglion of Meckel, being composed of *gray matter, must play an important part as a generator of nervous power*, of which, like a galvanic battery, it affords a continual supply; while the branches of the ganglion, under the influence of the diseased trunk, serve as conductors of the accumulated morbid nervous sensibility.

The bones of the cranium are liable to expansion, or thickening of their texture, from inflammatory action, most commonly dependent upon some constitutional taint. If the *os sphenoides* happened to be the seat of such disease, one or more of the foramina for the transmission of the nervous trunks might become very much contracted. A question might arise as to the effect of compression, from this cause, on the trunk of the second branch of the fifth pair, at the point where it is surrounded by the osseous sides of the *foramen rotundum.* From what has heretofore been stated, in relation to the law which governs the transmission of morbid sensibility along the trunk and branches of a nerve, subjected to an irritating cause, we should infer the supervention of neuralgia of the face, of the most severe character. In such a case, the operation of exsection of the trunk of the nerve, beyond the ganglion of Meckel, offers the best hope for relief; for, besides the removal of the trunk of the nerve, thus far, direct local depletion is obtained at the seat of the irritation; and, moreover, the portion of the nerve, placed in the foramen, will, most probably, become atrophied or diminished.

Pathological records corroborate the opinion which locates the seat of facial neuralgia on the nervous branches or trunks, after they have emerged from the ganglion of Gasser.

After the section of the fifth pair of nerves, within the cranium, it is a well established fact that the *general sensibility* is annulled in the superficial and deep parts of the face; and that the functions of the organs of *special sense* are disturbed. From this physiological fact, we arrive at the important diagnostic conclusion, that disease, involving the *trunk* of the fifth pair, and the ganglion of Gasser, so as to compromise its connections with the grand sympathetic, must be attended with pathological manifestations in the external organs of sense; the most remarkable of which are observed in the globe of the eye.

Cases illustrating this statement—important also in regard to the prognosis—are related by Herbert Mayo, Abercrombie, and others. The following case, published by M. Serres, (*Anatom. Comp. du Cerveau, etc.*,) is to the point: "A droite, l'insensibilité de la conjonctive était telle qu'on pouvait passer entre les paupières et le globe de l'œil les barbes d'une plume sans que le malade s'en aperçût; il-y-avait immobilité complète du globe de l'œil et de ses dépendances; la narine droite était également insensible à l'introduction d'un corps étranger; toutefois l'odorat n'avait pas complètement disparu. Le malade ne recevait aucune impression de l'application du sulfate de quinine sur la moitié droite de sa langue. Les gençives du même côté étaient molles, fongueses, noirâtres, détachées des os. Il-y-avait eu successivement inflammation de l'œil droit, coarctation de la pupille, opacité de la cornée et enfin perte de la vue. L'ouïe était diminuée à droite quelques jours avant la mort. *A l'ouveteure du cadavre, on trouva la cinquième paire ramolle à son origine ; jaunâtre et presque gélatiniforme. Cette altération s'enfonçait à une ligne ou deux dans la protubérance annullaire. Le ganglion de Gasser, de ce côté était d'une ligne et demie plus large que du côté sain ; il était jaunâtre. Quant à la petite racine du trijeaumeau, elle était intacte.*"

I shall now describe the operation I have recently adopted for the purpose of exsecting the trunk of the nerve beyond the ganglion of Meckel, as far as the foramen rotundum ; first recalling, that my first operation consisted in opening the *antrum Highmoranium* in front, with the crown of a trephine, and dissecting the nerve from before backward, towards the spheno-maxillary fossa.

*Operation.*—The trunk of the second branch of the fifth pair extends from the anterior part of the Gasserian ganglion to the place of its emergence, at the foramen infra-orbitale. It does not follow a direct line from before backward, in its course, but forms a curve, the concavity of which looks towards the mesial line. It may be divided into four parts, viz. 1st. That between the ganglion of Gasser and the posterior orifice of the foramen rotundum ; 2d. That embraced by the circumference of the foramen ; 3d. That which passes through the spheno-maxillary fossa ; and 4th. That which courses along the infra-orbital canal to emerge from the infra-orbital foramen.

The patient is laid upon the operating table, and complete anæsthesia effected by the administration of chloroform. The head, placed upon the sound side, and resting upon a solid cushion, with the face turned towards the operator, is supported firmly in this position, by an assistant detailed for this purpose. The other assistants are properly arranged, and the instruments, consisting of a small trephine, bistouries of various shapes, artery forceps, tenacula, bone forceps, the bone forceps of Lüer, several small chisels and a mallet, and other resecting instruments, are placed so as to be of easy access to the hand.

An incision is made, commencing opposite the spheno-maxillary fossa, upon the middle of the zygomatic arch, and extending forward and slightly downward, to a point a little below the foramen infra-orbitale. From the anterior extremity of this another incision is made downward, so as to divide entirely the tissues of the cheek and lip, midway between the median line and the commissure of the mouth. The soft tissues are now freely dissected from the malar and super-maxillary bones, and the nerve sought for as it emanates from the foramen infra-orbitale. This found, it is isolated from the other tissues, and the foramen and lower border of the orbit are completely exposed. The crown of a small trephine is then applied immediately below the infra-orbital foramen, and the antrum opened by removal of a portion of its anterior wall. This accomplished, with the chisel and Lüer's forceps, the lower part of the malar, and the outer portion of the superior malary bone connected with it, are removed as high upward as a line running horizontally forward, on a level with the lower border of the zygoma. The outer wall of the antrum is made bare of soft tissue, and with the bone forceps this wall is removed. The cavity of the antrum being now freely exposed, the nerve is detached from its upper wall from before backward, breaking down the wall of the infra-orbital canal, and carefully avoiding, at the same time, encroachment upon the soft tissues of the orbit.

It now remains to detach the portion of the nerve passing through the spheno-maxillary fossa with the ganglion of Meckel. At this stage of the dissection, the lower jaw must be held firmly and depressed by an assistant. The tissues lying upon the posterior wall of the antrum are separated from this part, and pushed backward by the finger and the handle of a scalpel. The spheno-maxillary fossa is now exposed, and the internal maxillary artery is seen sending off several branches, and is close related with the nerve. It is very necessary to avoid wounding this artery. By this time the trunk of the nerve is extensively detached, and it can be pulled downward so as to facilitate its isolation from the other tissues. The foramen rotundum must now be sought for. Its position can easily be ascertained by tracing with the finger the anterior border of the external pterygoid plate upward to its junction with the angle formed by the *body* and the *great ala* of the sphenoid bone. Proceeding inward from the upper part of this angle, for about two lines, the foramen rotundum is reached. With a blunt hook, such as is used in strabismus, the nerve is still further detached where it emerges from the foramen. Gentle traction is now used upon the trunk thus isolated, and grazing the surface of the sphenoid bone, with delicate blunt-pointed curved scissors, the nerve is severed at the base of the scull. The ganglion of Meckel can now be removed, or the branches descending to form it, not cut, can be divided.

In the early steps of the operation the bleeding is considerable, and the vessels must be at once secured. A pledget of lint is laid in the wound, and the lips of the incisions are brought together by points of the twisted suture.

In Forbes' case, the same external incisions were made as described in the operation just described. The stump of the nerve, remaining between the posterior part of the infra-orbital canal and the foramen rotundum, was sought for and found. It was then isolated from the surrounding tissues, and divided at the point of emergence from the foramen rotundum.

In conclusion, I embody my views in relation to aggravated neuralgia of the face in the following propositions:

I. That the second branch of the fifth pair, extending from the ganglion of Gasser to the foramen infra-orbitale, has two peripheries: one, formed by the terminal branches of the trunk, giving off along its course, the superficial parts of the face; the other, by the terminal branches emanating from the ganglion of Meckel.

II. That in cases of severe *tic douloureux*—the *dolor cru-*

*cians faciei* of Fothergill—the seat of the disease is in a portion of the trunk of the nerve, or in the entire trunk, between the ganglion of Gasser and the foramen infra-orbitale, including that part embraced by the foramen.

III. That the trunk of the nerve being injured or diseased, pain is felt at its periphery, as well as in the part morbidly affected.

IV. That impressions, acting upon the periphery of the nervous trunk, will be reflected upon the trunk, and give rise to paroxysms of neuralgic pain.

V. That the ganglion of Gasser, or the *common trunk* of the fifth pair, cannot be the seat of the disease, because experiments upon living animals, and pathological facts, derived from post-mortem examination, demonstrate that, when this ganglion and the trunk of the fifth pair are destroyed or injured, the eye of the corresponding side becomes destroyed from defective nutrition, and also that the other organs of *special* sense manifest symptoms of functional disturbance.

VI. That the encephalic strands of the fifth pair, on the cerebral side of *the common trunk*, cannot be the seat of the disease; as in such condition of the brain there would be symptoms denoting cerebral disturbance or disease, which never exist in tic douloureux.

VII. That division of the nerve externally to the foramen infra-orbitale, or anterior to the diseased portion of the trunk, will not effect a cure: because the point of disease being still left, the morbid sensibility is referred to the locality of the periphery, although that has been removed, or insulated.

VIII. That when only a portion of the trunk of the nerve is removed, anterior to the ganglion of Meckel, the remaining portion may become affected with the disease, and the symptoms be renewed with the same severity as before the operation.

IX. That the only operation which will cure the disease is the exsection of the trunk of the nerve on the cerebral side of the ganglion of Meckel ; because, 1st, the diseased part will thus be removed ; 2d, because the two peripheries of the nerve must thus be insulated from the encephalon ; 3d, because the influence of the ganglion of Meckel, in supplying morbid nervous sensibility, is destroyed ; 4th, because the sensibility of the two peripheries of the nerve is obliterated, and consequently external impressions cannot be reflected or transmitted.

X. That there is a possibility of the neuralgia returning for a time, even after the exsection of the trunk beyond the ganglion of Meckel, from disease attacking the small portion of the nerve still remaining in front of the ganglion of Gasser, or from pressure upon it, resulting from osteitis and contraction of the foramen rotundum ; the pain being referred, as already explained, to the original seat of the periphery.

XI. That, in such a case, however, the stump of the nerve, whether diseased or compressed by the circumference of the foramen rotundum, would be placed under circumstances leading to atrophy or resolution ; and that the disease, existing for a short time from such causes, would eventually subside.

XII. That the three trunks of the fifth or trifacial nerve, emanating from the ganglion of Gasser, and supplying in their aggregate the general sensibility to the face, when affected by neuralgia, are to be subjected, alike, to the same rules in regard to the etiology, pathology, and treatment. [*American Medical Gazette.*

